# Kissing tumors; the concurrent diagnosis of leiomyosarcoma and squamous cell carcinoma of the esophagus

**DOI:** 10.1186/s12893-020-01031-z

**Published:** 2021-01-06

**Authors:** Hamed Gheibollahi, Amirreza Dehghanian, Negar Taheri, Saeid Tavanafar, Seyede Sona Mousavi, Hamidreza Abbasi, Mohammadreza Sasani

**Affiliations:** 1 Oral and Maxillofacial Surgery, Department of Oral and Maxillofacial Surgery, Shiraz University of Medical Sciences, Shiraz, Iran; 2 Surgical and Clinical Pathology, Trauma Research Center, Shiraz University of Medical Sciences, Shiraz, Iran; 3 Molecular Pathology and Cytogenetics Ward, Department of Pathology, Shiraz University of Medical Sciences, Shiraz, Iran; 4 Department of Pathology, Shiraz University of Medical Sciences, Shiraz, Iran; 5 Trauma Research Center, Shahid Rajaee (Emtiaz) Trauma Hospital, Shiraz University of Medical Sciences, Shiraz, Iran; 6 Department of Radiology, Medical Imaging Research Center, School of Medicine, Shiraz University of Medical Sciences, Shiraz, Iran

**Keywords:** Esophageal neoplasms, Leiomyosarcoma, Esophageal squamous cell carcinoma

## Abstract

**Background:**

Esophageal leiomyosarcoma (LMS) is a rare tumor that constitutes less than 1% of all malignant esophageal tumors. Concurrent occurrence of esophageal leiomyosarcoma with squamous cell carcinoma (SCC) is even rarer than isolated leiomyosarcoma.

**Case presentation:**

In this report, we present a case of concurrent leiomyosarcoma and SCC in a 64-year-old woman presenting with vomiting and solid dysphagia, which has not been properly diagnosed following several referrals and diagnostic modalities. At last Exploratory laparotomy with gastric pull-up was performed in addition to radical laryngectomy with partial resection of the esophagus and subtotal thyroidectomy. Pathologic evaluation of the surgical specimen showed concurrent LMS (5.2 × 4.5 × 3 cm) and SCC (1.5 × 0.6 × 0.6 cm) at the same anatomical level in the proximal esophagus.

**Conclusions:**

This study proposes the importance of using ancillary diagnostic tests such as immunohistochemistry (IHC) to diagnose less common cases such as concurrent LMS and SCC.

## Background

Esophageal carcinoma is the eighth most common cancer worldwide with a poor prognosis due to its overly aggressive nature [1]. The two typical esophageal carcinoma forms, accounting for 95% of esophageal malignancies, include squamous cell carcinoma (SCC), arising from the squamous epithelial adenocarcinoma, affecting columnar glandular cells. Less than 1–2% of esophageal cancers are sarcomas, while other carcinomas, like melanomas, leiomyosarcomas, carcinoids, and lymphomas account for less than 1% of esophageal malignancies [2].

Leiomyosarcoma (LMS) is a high-grade slow-growing smooth muscle connective tissue tumor with late metastasis observed in different organs, such as the gastrointestinal tract and uterus.[3]. There are few case reports of esophageal LMS [4, 5]. There are even fewer cases reporting esophageal LMS with SCC, occurring/diagnosed simultaneously [6], or after esophageal SCC [7]. Some have also reported LMS and SCC's simultaneous incidence in the larynx [8] and oral cavity [9]. These reports refer to the possible concurrency of LMS and SCC in the upper aero-gastrointestinal tract. Nevertheless, the number of reported cases are very few, and there is much to be known in this regard. Here, we report a case of concurrent esophageal LMS and SCC anatomically located at the same level in a 64-year-old woman, presenting with vomiting and dysphagia, diagnosed and treated appropriately.

## Case presentation

**Fig. 1:**
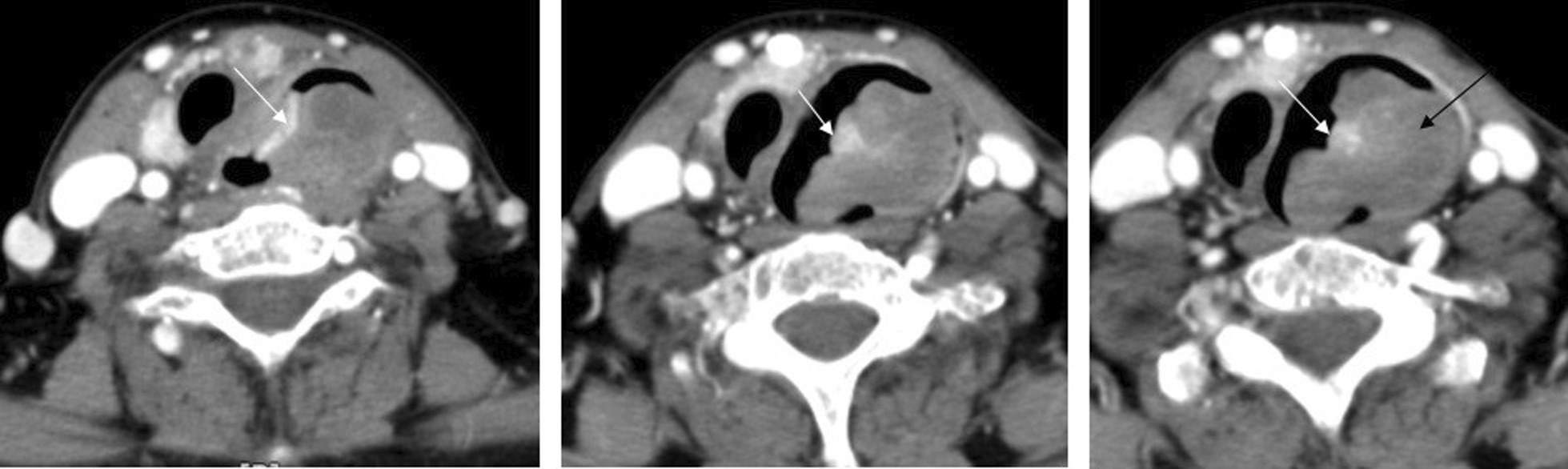
Post-contrast computed tomography images showing two lesions (hugging lesions) in the upper esophagus causing the tracheal shift to the right side: larger lesion (black arrow) is isodense with an area of the necrosis hugging the other small hyperdense lesion (white arrows) arising from the other side of the lumen

**Fig. 2:**
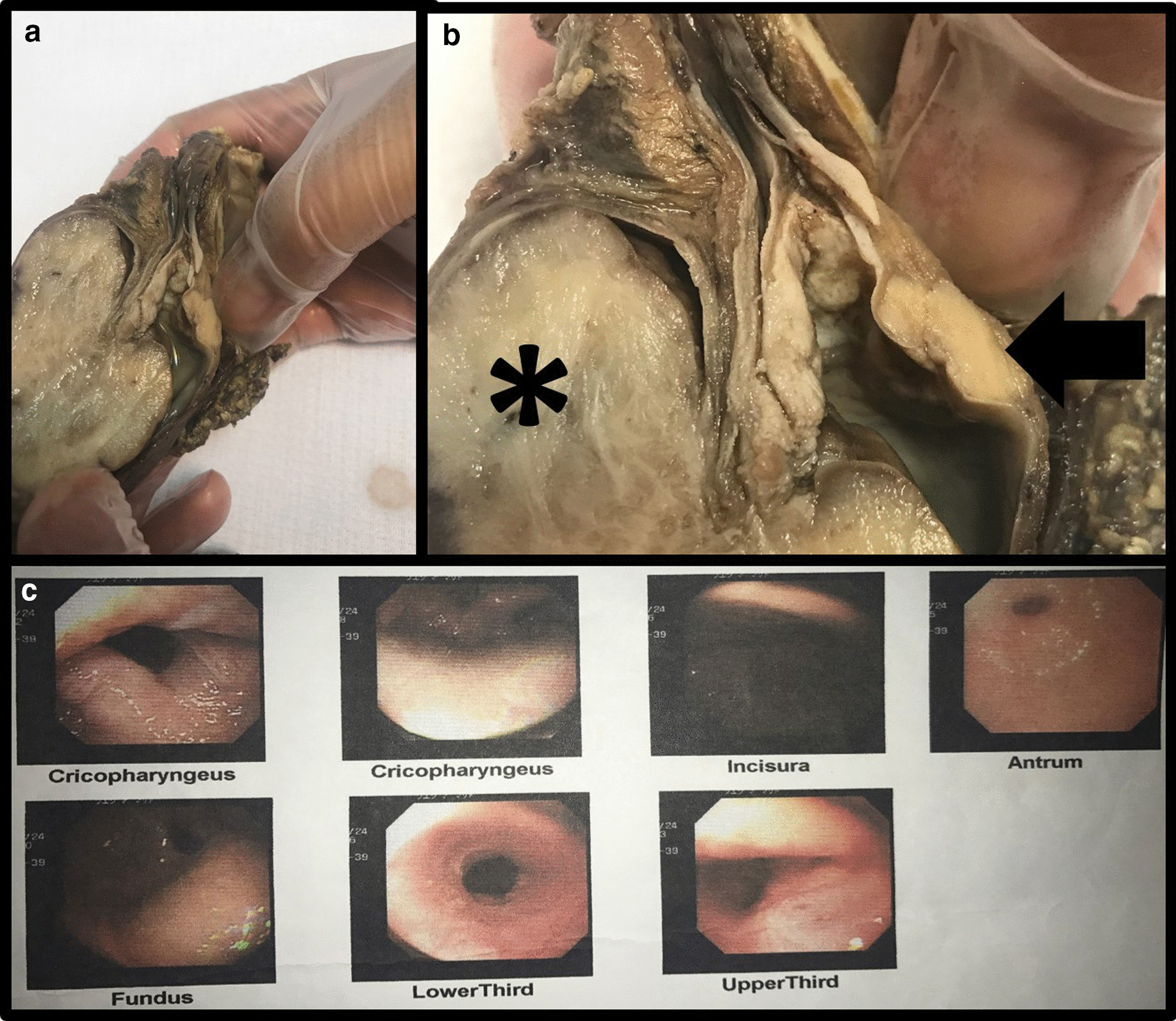
Gross and endoscopic findings of the patient's tumors. **a**, **b** Gross specimen of the resected tumors, which are located at the cricopharyngeal recess. The tumor diagnosed as leiomyosarcoma shows a smooth whitish glistening surface (asterisk), and the second tumor diagnosed as squamous cell carcinoma (arrow) is located in front of the first tumor that makes a stricture at the level of cricopharyngeal recess. **c** Endoscopic pictures at different levels show a stricture at the level of cricopharyngeal recess

The patient was a 64-year-old woman with seven children brought to the hospital with severe vomiting and a history of solid dysphagia; she had the symptoms for three months and was referred to different physicians, left without definite diagnosis before admission to our center.. Patient’s past medical history was unremarkable. Post-contrast computed tomography (CT) of the neck showed two lesions (kissing tumors) in the upper esophagus, causing the tracheal shift to the right side with no lymph node enlargement (Fig. 1). Physical examination revealed no abnormalities, and the results of laboratory examinations were within the normal range. CT-guided fine-needle aspiration biopsy evaluation of the mass showed a few pleomorphic spindle malignant cells among inflammatory cells in a bloody background. Additional endoscopy of the esophagus, stomach, and duodenum was performed for the patient, which showed a large tumor in the cricopharyngeus causing stricture, while the stomach and duodenum were normal. (Fig. 2).

The patient was scheduled for exploratory laparotomy with the exploration of cervical organs, resection of the pharynx, thyroid (partial), and esophagus and gastric pull up along with the insertion of a tracheostomy and bilateral chest tube, and cervical penrose.

### Surgical procedure

The patient was laid in a supine position. After preparation and sterilization, the abdomen was opened by a high midline incision. The surgeon inserted his finger into the mediastinum to assess the presence of any tumors. Stomach was mobilized by harmonic ligasure, lesser sac was opened, short gastric and left gastric arteries were ligated, and gastric pedicle was made on the right gastroepiploic arteries and epigastric area. Then, the esophagus was dissected bluntly by the finger in the mediastinum through the hiatus's opening and continued upward. Adhesion to the carina was found and released meticulously. Next, the collar incision was made on the neck. Then, subtotal thyroidectomy was done, and the specimen was sent for pathologic evaluation. Trocar was introduced in the sheath over the spine, and the esophagus was gently dissected and pulled up. The esophagus' blunt dissection was also carried out from the above until two hands could reach each other via the upper and lower incisions. After the esophagus' full release, resection of pharynx, larynx, and esophagus was done, and specimens of the proximal esophagus were sent for further pathological evaluation. Then, anastomosis of the mouth floor to the stomach was performed with polydioxanone suture 3/0, and the neck wound was irrigated and closed after insertion of a Penrose drain. The posterior part of the trachea was then fixed to the skin with Vicryl 3/0, and tracheostomy tube size 7.5 was inserted. The abdomen was irrigated, and the fascia was closed with a nylon loop one and irrigated. The skin was closed by nylon 3/0. Bilateral chest tubes No. 24 were inserted into the 5th intercostal space with a depth of 10 cm and fixed with silk 0 dressing. The surgery lasted for five hours and thirty minutes, and the patient received two packed cells and 6500 cc normal saline and had 4000 cc urination and 450 cc bleeding.

### Pathologic results

**Fig. 3:**
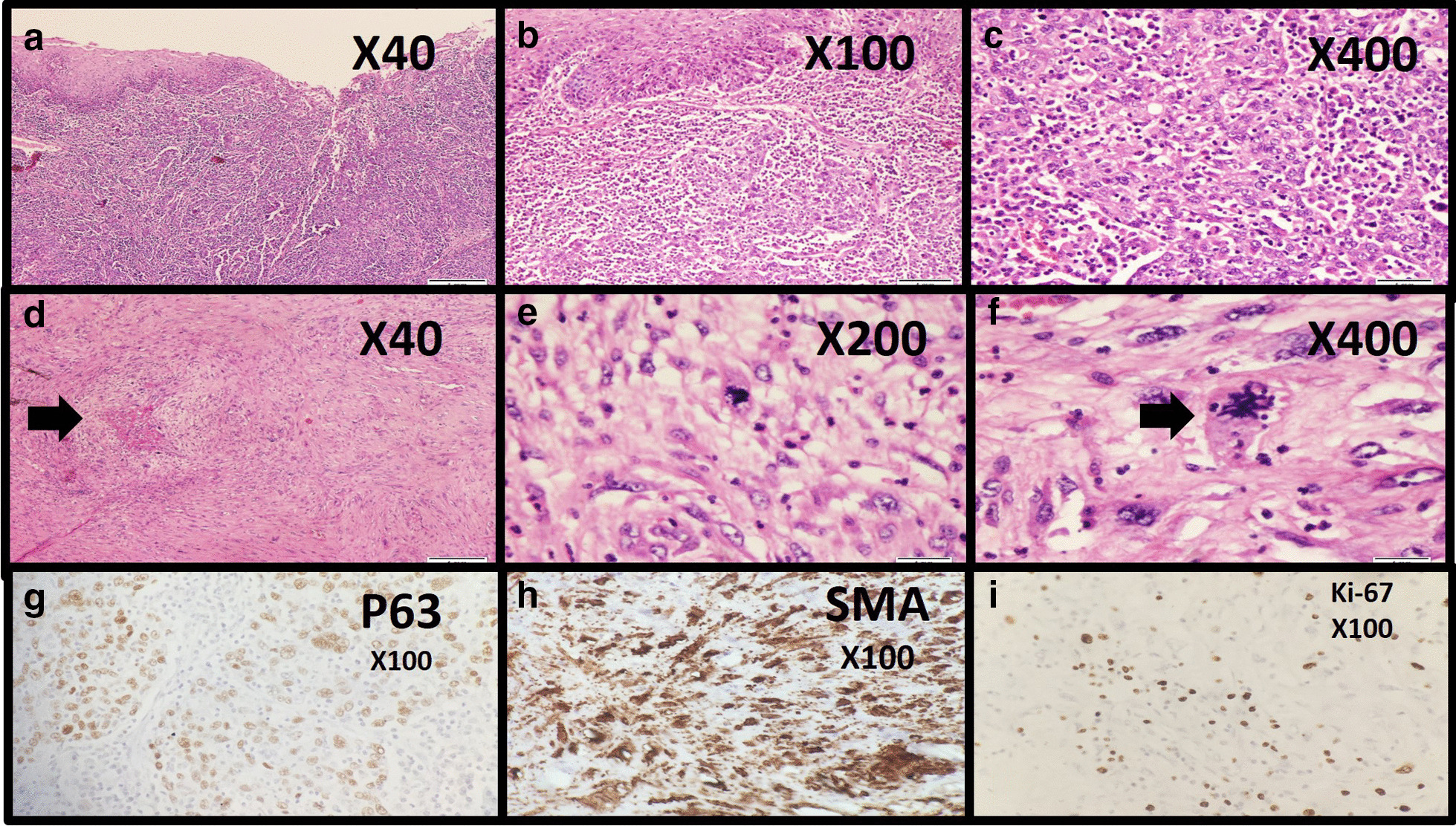
The histopathological examination results of the lesions; **a–c** show squamous cells growth with invasion to the lamina propria mucosa, muscularis mucosa, and submucosa. **d–f** Consisted of pleomorphic spindle cells with irregular nuclei and necrosis compatible with leiomyosarcoma (hematoxylin and eosin staining). **f** Shows 5–6 mitotic figures per 10 high-power fields, including atypical mitotic figures. **h** Shows the immunohistochemical staining results for leiomyosarcoma: positive for smooth muscle actin, and **i** show that the Ki-67 labeling index was 15%. **g** Shows the other tumor's immunohistochemistry study results, a positive result for P63 compatible with a diagnosis of esophageal squamous cell carcinoma. Tumors had no lymphovascular invasion

The gross pathological evaluation showed two separate grey cream masses with a smooth glistening surface at the cricopharyngeal level that made a stricture, as shown in Fig. 2. Microscopic evaluation showed malignant pleomorphic spindle cell neoplasm with high mitotic rate and many atypical mitoses in the larger mass (measuring 52 × 45 × 30) and invasive squamous cell nests proliferation in the smaller mass (measuring 15 × 6 × 6 mm). (Fig. 3) Immunohistochemistry (IHC) evaluation of the larger mass sections revealed negative results for P63, desmin, CD34, c-kit, DOG1, and cytokeratin (CK). It was diffusely positive for smooth muscle actin (SMA), S100, and also the high expression of proliferation index Ki67 to up to 15% was suggestive of malignant smooth muscle tumor. (Fig. 3) IHC evaluation of the smaller mass shows diffuse CK, P63 positivity (Fig. 3), and negative immunostaining for S100 and SMA was observed, which indicates the diagnosis of SCC. Tumors had no lymphovascular invasion, and the resection was curative with negative horizontal and vertical margins. Histopathological evaluation of the thyroid specimen shows nodular hyperplasia. Seven regional lymph nodes were free of metastasis.

The patient was discharged after ten days of hospital admission and was referred to the radio-oncologist. The patient underwent radiotherapy and was stable at six months' follow-up with no recurrence.

## Discussion

We presented a case with concurrent LMS and SCC in the proximal esophagus without invasion to other organs. Fortunately, the patient was appropriately treated with a radical laryngectomy, partial resection of the esophagus, and subtotal thyroidectomy. The case presented in this study had undergone several diagnostic investigations, including ultrasound, endoscopy, FNA, and pathologic examinations, leaving the patient without a definite diagnosis. As the presence of a tumor was suspected, we performed exploratory laparotomy for the patient with the exploration of cervical organs, resection of the pharynx, thyroid (partial), and esophagus in addition to gastric pull up along with the insertion of a tracheostomy and bilateral chest tubes, and cervical Penrose. This intervention was chosen considering that simultaneous resection of other solid organs and esophagectomy is considered to increase the chance of survival [10]. The specimens' histopathologic results showed that all organs, other than the proximal esophagus, such as distal margins of the esophagus, tracheal margin, larynx, epiglottis, and nuchal cord were free of tumor, and there was no need for further resection.

LMS is a rare tumor of the esophagus, and there are few similar cases reported in the literature [4, 5]. It is estimated that esophageal LMS accounts for about 0.5% of esophageal carcinomas [3]. Esophageal LMS concurrent with carcinoma is even rarer [6, 9, 11–16]. The summary of such cases is presented in Table 1. To our knowledge, there are only eight cases of simultaneous LMS and SCC, of which three cases were in the esophagus [6, 15, 16]. In a study by Jang et al. [6], a 72-year-old male patient presenting with chest pain was diagnosed with polypoid LMS in mid esophagus combined with SCC, similar to our case. However, the diagnostic method and tumor progression differed, as they reported a rapidly growing LMS, although LMS is generally considered a tumor with a slow growth rate [17]. Nakao et al. [7] also reported a 52-year-old Japanese man who developed esophageal LMS without metastasis four years after undergoing subtotal esophagectomy following the diagnosis of a well-differentiated SCC of the lower esophagus. We must insist that the currently presented case's novelty is the concurrent diagnosis of two separate LMS and SCC masses at the same anatomical level of the esophagus. There have also been few case reports of concurrent LMS and SCC in other organs near the esophagus, such as the larynx [8, 14] and oral cavity [9], that could propose a possible connection between LMS and SCC in the upper aerodigestive system.

**Table 1:** Summary of previous concurrent cases of leiomyosarcoma and squamous cell carcinoma

Authors and publication year	Location	Patients age and gender	Tumor size (cm)	Case description	Patients outcomes
Dorobisz et al. [11]	Larynx	75 years male	1.4 cm	Moderately differentiated squamous cell carcinoma was involving the subglottic area, the submucosal layer of the glottis on the right side, and the tracheal mucosa. Leiomyosarcoma was found in the subglottic area	To date, the patient has no recurrent lesions
Pongsuvareeyakul et al. [12]	Ovary	65 years female	17	Two distinct components of sarcomatous and invasive epithelial elements found in a mature cystic teratoma of the ovary	Dead due to unrelated cause after 1 month
Jang et al.[6]	Esophagus	72 years male	30 cm	The tumor consisted of pleomorphic spindle cells with mitosis and cell necrosis compatible with leiomyosarcoma Squamous severe dysplasia and focal stratified squamous epithelial invasion into the lamina propria were also noted compatible with squamous cell carcinoma	Two months' follow up showed no recurrence or distant metastasis
Kara et al. (two cases) [13]	Larynx	50 and 55 years males	Not mentioned	Case one: revealed spindle cell proliferation with mitotic figures. The squamous component of the tumor was well differentiated Case two: revealed leiomyosarcoma and squamous cell carcinoma on both vocal folds. Histological analysis showed spindle cell proliferation with mitotic figures, and a squamous component of the tumor was well differentiated	Case one: no recurrent lesion at 3 years follow up Case two: no recurrent lesion at 15-month follow up
Tomidokoro et al. [14]	Larynx	74 years male	left vocal fold was 1.2 × 0.9 × 0.8 cm right vocal fold was 1.2 × 0.8 × 0.7 cm	The left vocal fold tumor was moderate to poorly differentiated SCC The polypoid tumor of the right vocal fold was diagnosed as LMS	No recurrences at 9-month follow-up
Dios et al. [9]	Oral Cavity	67 years male	1.5 cm on the soft palate and cobblestone area on lateral tongue	Soft palate lesion was diagnosed as LMS, and tongue lesion was diagnosed as SCC	LMS has no recurrence, but tongue SCC recurred at two and 19 months after the first surgery
Eroğlu et al. [15]	Esophagus	46 years male	3 cm	The tumor was composed of spindle cells with eosinophilic cytoplasm and fusiform nuclei organized into bundles. Pleomorphic and giant cells and mitoses were present. Squamous cell carcinoma and leiomyosarcoma combination was reported	No recurrence at 16 months follow up
Gaede et al. [16]	Esophagus	56 years male	8.5 × 6 × 3 cm, second tumor adjacent but distinctly separate was 2 cm mass with similar configuration	The lesion was diagnosed as LMS The second lesion showed marked atypia of the epithelium's entire thickness, indicating squamous cell carcinoma in situ, with foci of micro invasion	Unknown

As esophagus LMS symptoms are not different from other esophageal carcinomas and patients present mainly with dysphagia and/or chest pain [4], more accurate diagnostic methods are required for diagnosis. In our case, several modalities such as ultrasound, endoscopy, and FNA could not result in a definitive diagnosis, and we had to perform exploratory surgery for resection of the organs suspected of tumor involvement. Jang and colleagues suggested using the Positron emission tomography/computed tomography scan as a useful tool in diagnosing esophageal LMS [6]. Nakao et al.[7] also performed IHC examination that showed positive SMA and vimentin and negative CK and S100, which is similar to the present study showing positive results for SMA and Ki67 expression in LMS.

## Conclusion

By presenting this rare case of concurrent esophageal LMS and SCC, the authors aimed to highlight the concurrency of such a diagnosis that has a high probability of being misdiagnosed in a patient. We must also propose that such cases urge ancillary diagnostic tests such as IHC in the better evaluation of a patient's tumor and lead to a more definite and proper diagnosis.

## Data Availability

Not applicable.

## Abbreviations

SCC: Squamous cell carcinoma
LMS: Leiomyosarcoma
FNA: Fine-needle aspiration
IHC: Immunohistochemistry
CT: Computed tomography
